# A Metallic Screw Gun-Traumatic Vertebral Artery Dissection Injury: A Case Report and Literature Review

**DOI:** 10.7759/cureus.110396

**Published:** 2026-06-07

**Authors:** Abdulaziz Y Alahmed, Osama A Barnawi, Abdullah S Alali, Zainab B Alsalman, Jehad Ahmed

**Affiliations:** 1 Neurosurgery, Aseer Central Hospital, Abha, SAU; 2 College of Medicine, King Faisal University, Al-Ahsa, SAU; 3 Neurosurgery and Spine, Aseer Central Hospital, Abha, SAU

**Keywords:** cerebral digital substraction angiography, foreign body removal, skull bas and vascular neurosurgery, traumatic vertebral artery dissection, vertebral artery (va)

## Abstract

Traumatic vertebral artery dissection (VAD) is a tear of the intimal layer, which may extend into the tunica media, resulting in blood tracking within the arterial wall and consequent luminal stenosis. It presents complex challenges for surgical and neurovascular management. The most commonly affected vertebral artery segments are V2 and V3, due to their anatomical vulnerability to mechanical stress during neck motion. Potential complications include ischemic stroke, thromboembolism, subarachnoid hemorrhage, and pseudoaneurysm, which necessitate early diagnosis and timely intervention using endovascular or surgical approaches. We report the case of a 32-year-old previously healthy male who sustained a penetrating cervical injury from a high-speed screw gun. On presentation, he was fully conscious and neurologically intact, complaining of neck pain and restricted range of motion. Initial computed tomography (CT) and computed tomography angiography (CTA) demonstrated a metallic foreign body at the level of the C1-C2 vertebrae involving the right vertebral artery, without evidence of cerebral ischemia. Digital subtraction angiography (DSA) revealed occlusion of the V2 segment of the right vertebral artery with preserved distal flow. Following multidisciplinary discussion, a consensus was reached to proceed with balloon occlusion testing, right vertebral artery coil embolization, and subsequent surgical removal of the foreign body via a suboccipital craniotomy in a staged manner. The postoperative course was uneventful, and the patient was discharged neurologically intact on antiplatelet therapy for secondary stroke prevention. This case illustrates a rare mechanism of traumatic VAD with a favorable clinical outcome. It highlights the critical role of early vascular imaging, multidisciplinary collaboration, and individualized endovascular and surgical management in penetrating vertebral artery injuries.

## Introduction

The incidence of traumatic vertebral artery (VA) dissection is estimated at 1-5 per 100,000 in the general population, making it a rare condition encountered by neurosurgeons and spine surgeons [1]. Anatomically, the vertebral artery is divided into four segments: prevertebral (V1), foraminal (V2), atlantic (V3), and intracranial (V4) [2]. The V2 and V3 segments demonstrate a higher prevalence of dissection compared to the other segments, with the V3 segment being more commonly affected due to its anatomical susceptibility to mechanical stress during neck rotation and extension [3]. Following traumatic vertebral artery dissection, complications such as thromboembolic stroke, subarachnoid hemorrhage, and pseudoaneurysm may occur. Cross-sectional angiographic imaging, including computed tomography angiography (CTA) and digital subtraction angiography (DSA), plays a crucial role in establishing the diagnosis and identifying associated vascular complications [4]. In patients with vertebral artery dissection (VAD), the primary management focus is stroke prevention and maintenance of adequate cerebral perfusion. Surgical or endovascular intervention is infrequently required, as most patients are successfully managed with conservative treatment [5].

## Case presentation

A 32-year-old male with an unremarkable medical history presented to the emergency department following a high-speed screw gun injury. On arrival, the patient was conscious, alert, and oriented. His Glasgow Coma Scale score was 15/15, with no neurological deficits. He demonstrated only a reduced range of motion during lateral neck rotation. A clear entry wound was noted in the left submandibular region. Plain radiography and non-contrast computed tomography of the brain and cervical spine were performed (Figures 1-4).

**Figure 1:**
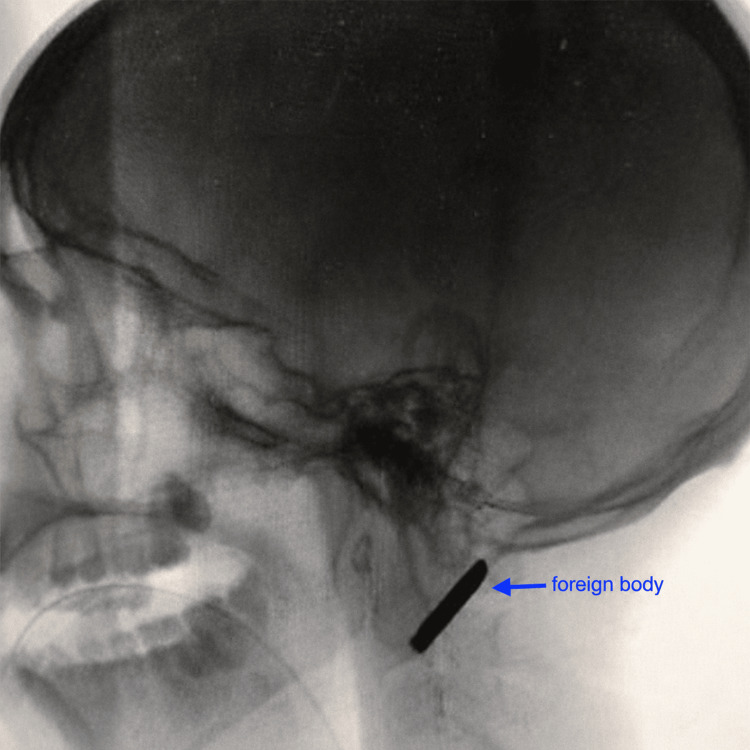
X-ray image, lateral view. Lateral X-ray of the cervical spine demonstrates a radiopaque object at the level of the C2 vertebral body (blue arrow).

**Figure 2:**
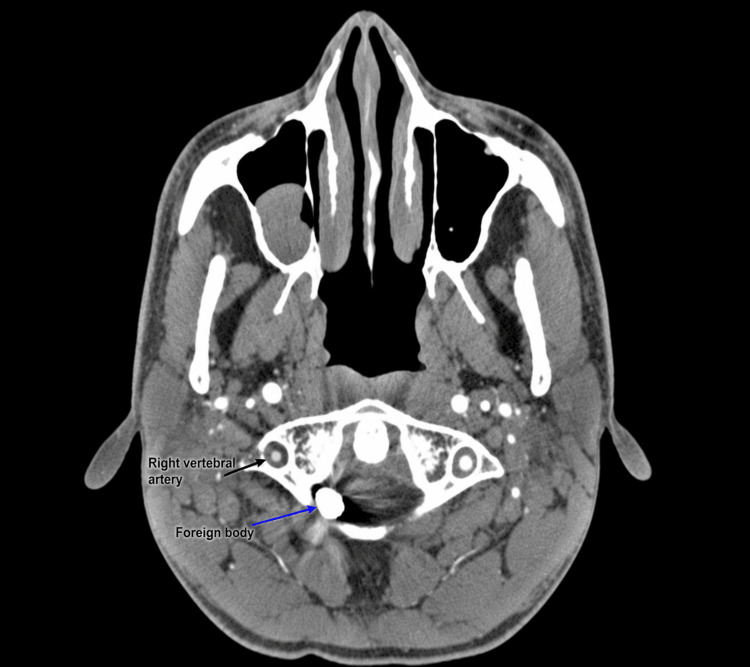
Non-contrast cervical CT with axial view and bone window. A metallic foreign body at the C1–C2 level (blue arrow) obscures the right vertebral artery at the site of its exit from the C2 foramen transversarium.

**Figure 3:**
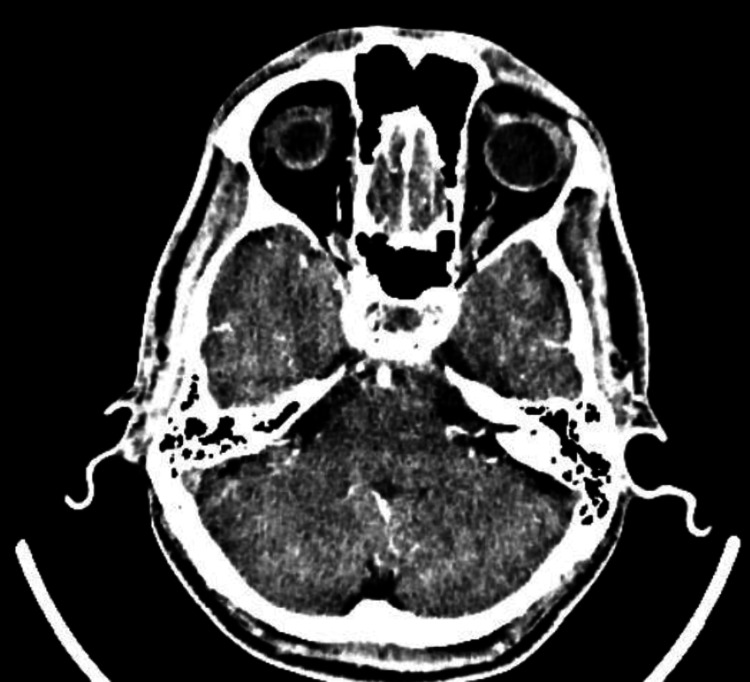
CT angiography of the brain. No evidence of vascular occlusion, significant stenosis, or acute vascular abnormality was identified within the posterior circulation on CTA of the posterior fossa.

**Figure 4:**
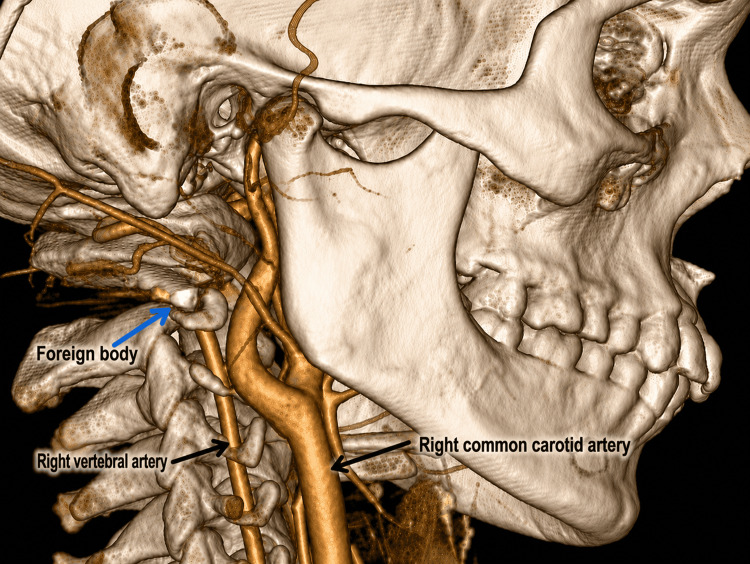
A 3D CT brain reconstructed image. A three-dimensional reconstructed CT image clearly demonstrates a metallic object traversing the vertebral artery exit and abutting its outer layer at the level of the right foramen transversarium (blue arrow).

A multidisciplinary discussion was held with the endovascular team, and it was agreed to perform a balloon occlusion test (BTO) of the right vertebral artery prior to surgical exploration after the diagnosis of right vertebral artery dissection (VAD) had been confirmed by digital subtraction angiography (DSA), the gold standard modality.

Under local anesthesia, a compliant balloon microcatheter was advanced and inflated under fluoroscopic guidance until complete occlusion of antegrade flow in the right vertebral artery was achieved. Serial neurological examinations were performed over a 30-minute monitoring period. At the end of the test, the patient did not demonstrate any transient or permanent neurological deficits, indicating adequate collateral circulation through the left vertebral artery to support the posterior circulation.

Based on the results of the balloon occlusion test, the right vertebral artery was sacrificed via coil embolization to reduce the risk of future thromboembolic ischemic events (Figures 5-7).

**Figure 5:**
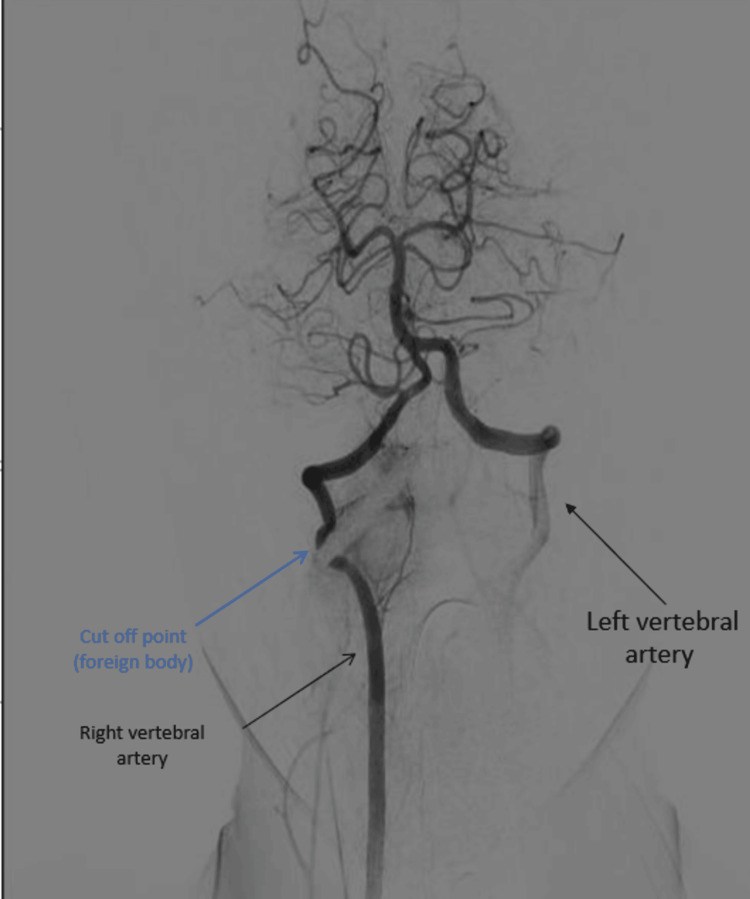
DSA of the right vertebral artery. Shows injection of the right vertebral artery demonstrating obliteration at the V2 segment (blue arrow) with patent flow beyond this site. There is also clear and efficient collateral contrast flow through the left vertebral artery, demonstrating adequate perfusion from the contralateral side of the dissection. DSA: Digital subtraction angiography

**Figure 6:**
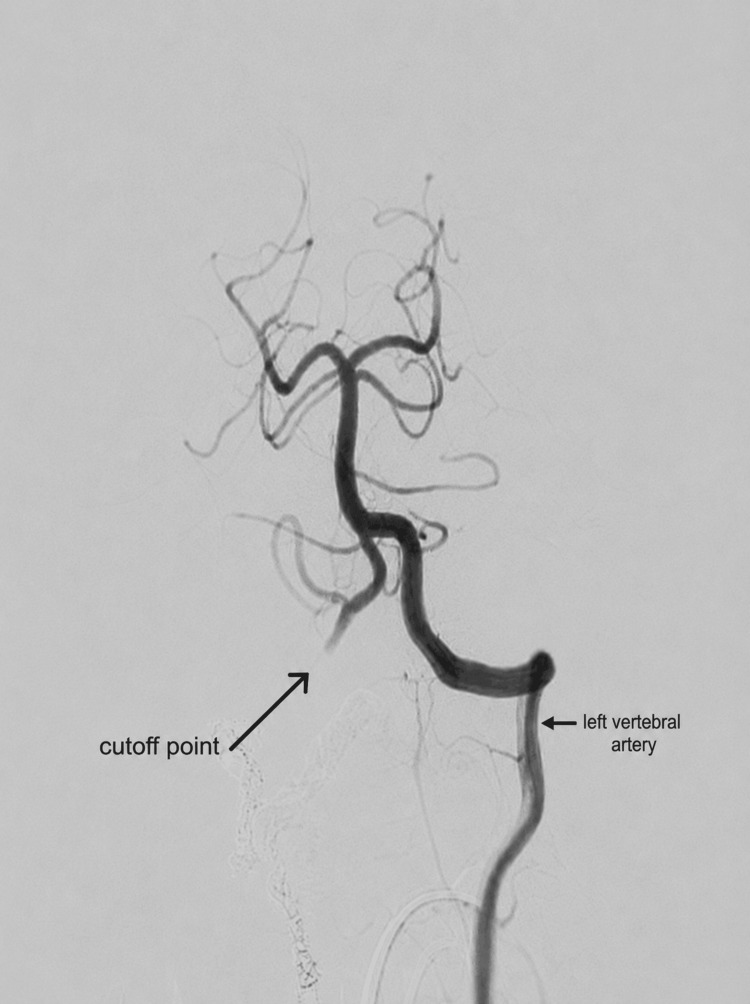
DSA of the left vertebral artery. Injection of the left vertebral artery demonstrates cut-off of contrast flow in the contralateral vertebral artery, with patent flow beyond the site of obstruction. This confirms adequate flow through the left vertebral artery and supports the diagnosis of right vertebral artery dissection (VAD). DSA: Digital subtraction angiography

**Figure 7:**
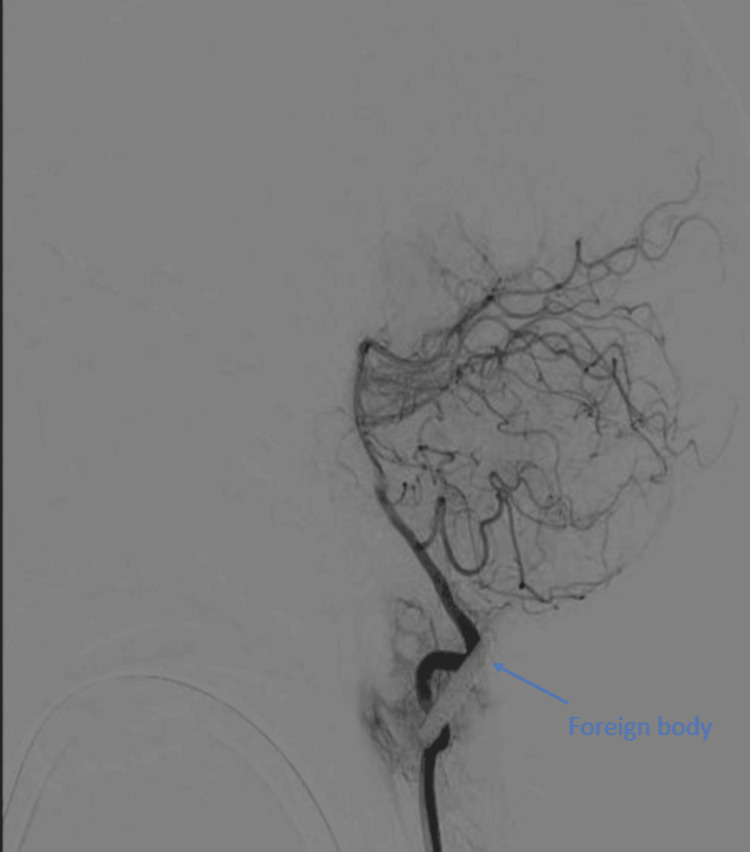
DSA of the right internal carotid artery. Shows injection of the right internal carotid artery demonstrating obliteration of contrast flow (blue arrow) from the C3–C7 segment, with patent flow beyond this site. Injection of the right carotid artery was performed to ensure patency of flow, as the foreign body was located on the right side of the neck. This contrast obliteration was determined to be an artifact caused by the presence of the foreign body on the same side as the vertebral artery dissection (VAD), which was confirmed by cervical CTA and intraoperative findings. DSA: Digital subtraction angiography

Following coil embolization and foreign body removal (Figures 8, 9), the patient was transferred to the intensive care unit (ICU) for close neurological and hemodynamic monitoring. He was subsequently discharged without any neurological deficits and was prescribed aspirin for six months for stroke prevention, in accordance with American Stroke Association recommendations. The patient was followed up at three-month intervals for one year and remained asymptomatic, with no new neurological symptoms or ischemic events.

**Figure 8:**
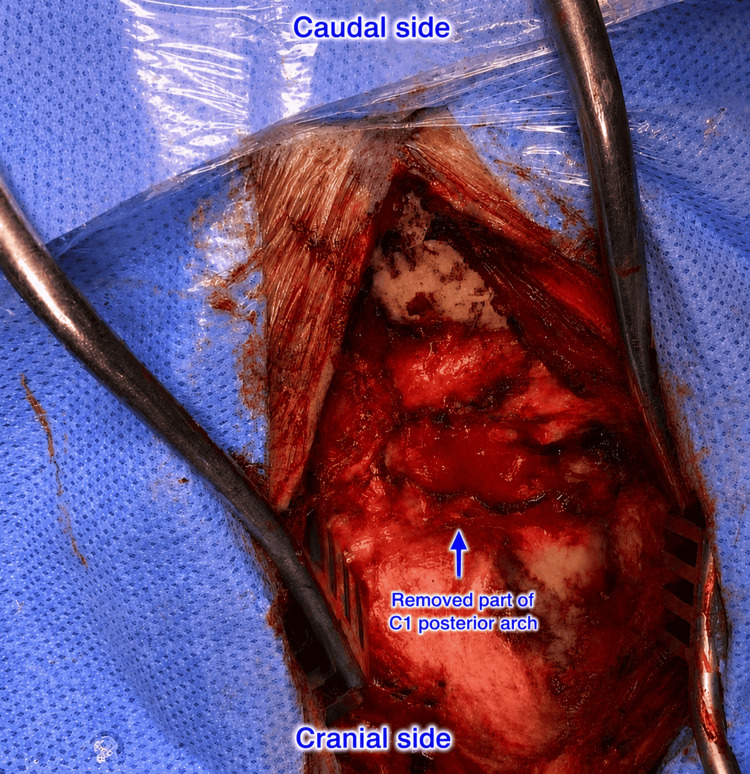
Intraoperative view. The patient underwent foreign body removal via a suboccipital approach for C1 and C2 exploration, accompanied by removal of the C1 posterior arch for better visualization of the right C2 foramen transversarium. The patient was subsequently transferred to the recovery area without any postoperative complications.

**Figure 9:**
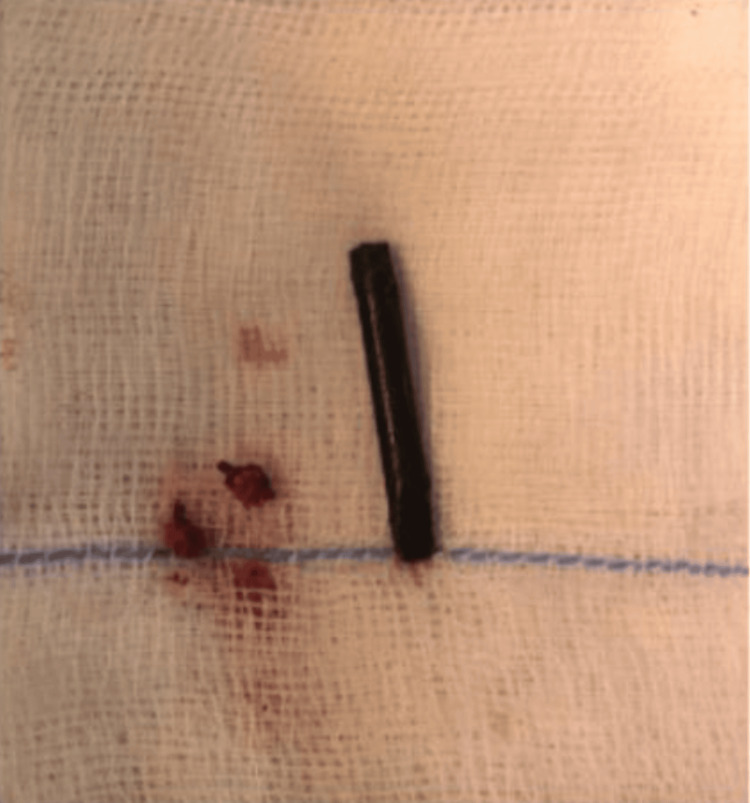
Metallic foreign body after removal.

## Discussion

VAD is usually classified into spontaneous and traumatic injuries. Spontaneous dissection is associated with intrinsic factors, such as Ehlers-Danlos syndrome, which increases the predisposition to dissection compared to the general population. In contrast, traumatic dissection is usually precipitated as a sequela of trauma, including sports injuries, massage, chiropractic manipulation, and even rapid neck rotation [6]. Spontaneous and traumatic VAD are both considered epidemiologically uncommon compared to carotid artery dissection, accounting for 20% and less than 1% of cerebrovascular dissecting events, respectively. Cervical fractures leading to VAD account for 27.5% of cases as a sequela of road traffic accidents, while gunshot or penetrating trauma is considered very rare, ranging from 1% to 6% of all cases. The majority of cases are iatrogenic, involving both anterior and posterior surgical approaches [7,8]. In our case, the mechanism of injury is considered rare because of its high kinetic energy and the remarkable prognosis.

The type of trauma may influence the predilection for vertebral artery segment involvement, making the V2 segment the second most commonly affected due to its length, which increases its vulnerability to injury following fractures. In addition, it has a greater surface area exposed to penetrating and gunshot injuries. This is preceded by the V3 segment, which is the most commonly involved because of its greater mobility [3,9].

A systematic review of 1,927 patients with spontaneous and traumatic VAD demonstrated that the most common presenting symptoms were headache, dizziness or vertigo, and neck pain [10]. Other expected symptoms are related to the vascular territory involved. Due to hypoperfusion, patients may present with Horner syndrome, Wallenberg syndrome, internuclear ophthalmoplegia, cerebellar symptoms, lower cranial nerve palsies, Brown-Sequard syndrome, cervical myelopathy, or radiculopathy. Moreover, blood flow stagnation may lead to a thromboembolic stroke [3,11]. Furthermore, a study of 154 patients revealed that only 21% of traumatic VAD cases were symptomatic or presented with ischemic-like symptoms [12].

When a patient presents to the emergency department with suspected traumatic VAD, CTA is the preferred initial imaging modality because of its rapid acquisition time and the importance of the clinical decisions based on its findings. CTA can identify the degree of stenosis and the presence of pseudoaneurysm. In addition, it may suggest the presence of an intramural hematoma by demonstrating a discrepancy in vertebral artery size compared to the contralateral side. Finally, CTA serves as a bridging tool prior to DSA [13].

Magnetic resonance imaging (MRI) is another imaging modality used when the patient exhibits clinical features suggestive of acute ischemia, such as weakness or sensory deficits. Four MRI sequences are commonly utilized. The T1 sequence can identify intramural hematoma, which appears as subintimal hyperintense signals. Most importantly in emergency settings, diffusion-weighted imaging (DWI) and apparent diffusion coefficient (ADC) sequences are used to identify ischemic areas. Due to signal restriction, ischemia appears hyperintense on DWI and hypointense on ADC, forming the so-called “combo sign,” which is considered the gold standard for identifying acute ischemic stroke [14]. Magnetic resonance angiography (MRA) demonstrates findings similar to CTA. However, CTA remains the superior modality in terms of time and cost unless MRI is specifically indicated because of inconclusive CTA findings [15].

Ischemic stroke is typically excluded using two standard imaging modalities: CT perfusion studies or MRI with diffusion-weighted imaging (DWI) and apparent diffusion coefficient (ADC) sequences. However, in our institution, ischemic stroke in this case was ruled out clinically. This approach was supported by imaging with computed tomography angiography (CTA) followed by digital subtraction angiography (DSA). The decision to rely on these modalities was influenced by the unavailability of CT perfusion studies at our center, as well as the patient’s refusal to undergo urgent MRI with DWI and ADC sequences because of financial constraints. Importantly, from the initial presentation, the patient exhibited no neurological deficits, which further supported the clinical exclusion of ischemic stroke. Therefore, CTA was deemed sufficient for the initial evaluation, and management subsequently continued with DSA.

There are many indications for balloon test occlusion (BTO), all of which are aimed at determining whether the vertebral artery can be sacrificed. The goal of this test is to assess the patency, adequacy, and flow of the contralateral side in maintaining vertebrobasilar circulation following permanent closure, if required. Indications include traumatic VAD, vertebral artery fusiform aneurysm, and trauma-associated pseudoaneurysm. In our case, we planned to coil the site of dissection and trauma; therefore, BTO was performed on the ipsilateral side. Fortunately, the left vertebral artery was able to maintain adequate posterior circulation perfusion [16].

The primary goals in traumatic VAD management are stroke prevention and preservation of neurological function. Traumatic VAD associated with acute ischemic stroke within the 4.5-hour therapeutic window is managed with thrombolysis. A meta-analysis demonstrated reassuring findings, showing that thrombolytic therapy does not increase the risk of intramural hematoma, despite previous theoretical concerns [17]. The American Stroke Association recommends endovascular stenting only in cases of recurrent ischemic stroke despite preventive medical therapy [18].

Because of the high rate of recurrent bleeding within the first 24-72 hours following intracranial vertebral artery dissection (V4 segment), urgent management, either surgical or endovascular, is preferred [19]. Coil embolization has a high success rate and a low recanalization rate (2.74%), especially in the absence of posterior inferior cerebellar artery (PICA) involvement or contralateral vertebral artery hypoplasia [20]. Stent-assisted coiling is used as an emergent treatment when sac dilatation is present to improve embolization and prevent rupture or rebleeding [21]. Microsurgical bypass with adjunctive coiling may also be considered; however, important limitations include anatomical variations, patient clinical status (Hunt and Hess score), and, most importantly, neurosurgical expertise [22]. In our case, the priority was securing the dissection site for neurofunctional protection, after which the patient was transferred immediately to the operating room for foreign body removal. Prioritization of endovascular coiling was based on the possibility of embolic migration and the vulnerability of the vessel to rupture and bleeding.

The use of antiplatelet agents versus anticoagulants for thromboembolic stroke prevention remains controversial; however, consensus has been reached regarding treatment duration, which is recommended to be 3-6 months according to the American Stroke Association [23]. A meta-analysis demonstrated no significant difference between antiplatelet agents and anticoagulants in preventing future stroke, with reported risks of 2.6% and 1.8%, respectively [24].

Generally, the outcome following extracranial traumatic VAD is excellent. A literature review demonstrated that 50% of patients had no neurological deficits, while 21% and 25% experienced mild and moderate deficits, respectively. Only 4% of cases resulted in mortality [24]. Initial presentation with neurological deficits has a detrimental effect on prognosis, as 40% of these patients may develop permanent deficits without significant recovery, and mortality may reach 10% in cases with intracranial extension. However, a case report of a 21-year-old female with left-sided VAD who presented with left-sided incoordination and visual field defects demonstrated complete recovery 10 months after dissection onset, despite an MRI performed eight months later showing multiple mature infarcts in the cerebellum and occipital lobes. This highlights the importance of additional prognostic factors such as age [25,26].

In contrast, intracranial traumatic VAD carries a poorer prognosis because of its higher predisposition to rupture and subsequent subarachnoid hemorrhage. Recurrent dissection carries a very low risk, accounting for only 2% of cases, while only 1.4% of patients experience recurrent stroke [25,27].

## Conclusions

Traumatic vertebral artery dissection (VAD) is an uncommon condition that may occur following penetrating or high-energy cervical trauma and may be associated with clinically significant complications. This case highlights that favorable neurological outcomes can be achieved through early recognition, prompt vascular imaging, and a coordinated multidisciplinary approach, even in the presence of significant vascular injury. Digital subtraction angiography (DSA) remains a key modality for definitive diagnosis and procedural planning. Management should be individualized according to the clinical and anatomical findings and may include endovascular treatment, surgical exploration, and antithrombotic therapy to reduce the risk of ischemic and hemorrhagic complications. In this case, a combined surgical and endovascular approach was associated with good clinical recovery.
